# Antimicrobial Photodynamic Therapy in the Control of *Pseudomonas syringae* pv. *actinidiae* Transmission by Kiwifruit Pollen

**DOI:** 10.3390/microorganisms8071022

**Published:** 2020-07-10

**Authors:** Margarida M. Lopes, Maria Bartolomeu, Ana T. P. C. Gomes, Etelvina Figueira, Ricardo Pinto, Luís Reis, Victor M. Balcão, M. Amparo F. Faustino, M. Graça P. M. S. Neves, Adelaide Almeida

**Affiliations:** 1 CESAM, Department of Biology, University of Aveiro, 3810-193 Aveiro, Portugal; margaridamlopes@ua.pt (M.M.L.); maria.bartolomeu@ua.pt (M.B.); ana.peixoto@ua.pt (A.T.P.C.G.); efigueira@ua.pt (E.F.); rl.pinto@ua.pt (R.P.); victor.balcao@prof.uniso.br (V.M.B.); 2 APK-Associação Portuguesa de Kiwicultores, 4520-249 Santa Maria da Feira, Portugal; bioearth@hotmail.com; 3 PhageLab-Laboratory of Biofilms and Bacteriophages, University of Sorocaba, 18023-000 Sorocaba/SP, Brazil; 4 LAQV-REQUIMTE, Department of Chemistry, University of Aveiro, 3810-193 Aveiro, Portugal; faustino@ua.pt

**Keywords:** Antimicrobial photodynamic therapy, photosensitizers, bacterial infection, *Pseudomonas syringae* pv. *actinidiae*, kiwifruit pollen

## Abstract

*Pseudomonas syringae* pv. *actinidiae* (Psa) is a phytopathogen responsible for bacterial canker in kiwifruit plants and can be disseminated through pollen. This study aimed to evaluate the effectiveness of antimicrobial photodynamic therapy (aPDT) in the inactivation of Psa on kiwifruit pollen using New Methylene Blue (NMB) and Methylene Blue (MB) in the presence/absence of potassium iodide (KI). Pollen germination assays were also performed to evaluate if it was affected by aPDT. Higher reduction of Psa was achieved using NMB (5.0 μM) combined with KI (100 mM) in vitro (ca. 8 log CFU mL^−1^ after 90 min of irradiation), while NMB alone promoted a lower reduction (3.7 log CFU mL^−1^). The most efficient NMB concentration with KI was used to study the photodynamic efficiency of MB (5.0 μM). MB with KI photo-inactivated Psa more efficiently than NMB, causing the same bacterial reduction (ca. 8 log CFU mL^−1^) in half the irradiation time (45 min). Therefore, MB was selected for the subsequent ex vivo aPDT assays in pollen. Almost all the Psa cells added artificially to the pollen (3.2 log CFU mL^−1^) were photo-inactivated (3.1 log CFU mL^−1^), whereas aPDT had a low effect on pollen natural microorganisms. When KI was added, a significant increase in aPDT effectiveness was observed (4.5 log CFU mL^−1^). No negative effects were observed in the pollen germination after aPDT. The results show aPDT is an effective and safe method to Psa inactivation on kiwifruit pollen, and MB use is a promising alternative in the control of Psa transmission.

## 1. Introduction

*Pseudomonas syringae* pv. *actinidiae* (Psa) is a phytopathogen and the causal agent of bacterial canker of both green-fleshed kiwifruit (*Actinidia deliciosa*) and yellow-fleshed kiwifruit (*Actinidia chinensis*), resulting in massive damages in orchards [1] and causing severe economic losses worldwide [2,3]. Psa was first isolated, identified, and described in Japan in 1984 [1]. Afterwards, until 2008, Psa was also found in China, Italy, and South Korea [2,4,5]. However, more recently, Psa has re-emerged, causing destruction of the main areas of kiwifruit production worldwide, being considered as a pandemic disease [2,3,4].

Presently, there are six genetically different biovars of Psa spread all over the world [2], varying in their aggressiveness to the plant. Biovar 3 is highly pathogenic and is affecting kiwifruit orchards in several countries worldwide (New Zealand, Italy, France, Spain, Portugal, and China) [2,6], being called “Psa-V” (from virulent) due to its high aggressiveness [6,7].

Psa control and treatment is a challenge. The approved treatments for this disease consist of spraying the orchards with copper derivatives and/or antibiotics, but such treatments are highly toxic to both humans and the environment and may lead to the development of bacterial resistance [8,9]. Antimicrobial photodynamic therapy (aPDT) can be a promising alternative, allowing to surpass the negative impacts of the current treatments. 

aPDT requires the combination of three components: a photosensitizer (PS), visible light, and molecular oxygen, resulting in the production of reactive oxygen species (ROS), which affect lipids, proteins, and nucleic acids [10,11,12,13], causing irreversible damages in microorganisms. The multitarget nature of aPDT minimizes the risk of bacterial resistance development, which provides an advantage over conventional treatments [9,14,15,16,17,18].

aPDT has been tested in the control of plant diseases caused mainly by fungi [19,20,21,22], but only very recently aPDT application in the control of Psa-induced kiwifruit canker was reported by our research group [9,23], without negative effects on the leaves of kiwifruit plants. The results show that aPDT mediated by cationic porphyrins was able to successfully inactivate Psa in both in vitro and in ex vivo (on kiwifruit leaves) conditions [9,23].

Kiwifruit pollen has been proven to be responsible for the dissemination of Psa among orchards [24]. However, few efforts have been taken to efficiently inactivate Psa on the pollen to control its dissemination. In fact, there is no approved treatment yet to efficiently inactivate Psa on the kiwifruit pollen. The use of heat as a potential method to inactivate the Psa present on the pollen has been studied [25]. The authors found that the combination of 35 °C with relative humidity at 50% or less, during more than 20 h, was the treatment conditions with more potential, thus far, to control the Psa without loss of pollen viability. According to the Portuguese Association of Kiwifruit growers (APK, Associação Portuguesa de Kiwicultores), kiwifruit pollen stored at −20 °C up to 3 years preserves its viability and also avoids the multiplication of Psa, which is the only measure that is taken to control Psa on pollen. Moreover, assisted pollination is increasing worldwide. In Portugal, for example, assisted pollination currently already represents 10–15% of the kiwifruit production (APK). Once assisted pollination uses acquired pollen, which is very expensive (2000–3500 €/kg), it is of extreme importance to develop an effective method to ensure the absence of Psa on sold pollen and at the same time maintaining its viability. 

In the present study, the inactivation of Psa was first studied in vitro using two well-known phenothiazine derivatives, New Methylene Blue (NMB) and Methylene Blue (MB), in the presence and the absence of potassium iodide (KI) to find the best conditions to photo-inactivate Psa. Then, the efficiency of aPDT to control Psa contamination on kiwifruit pollen was studied using the best conditions found. Pollen germination was also quantified to evaluate the pollen viability following the aPDT treatment. The in vitro assays were performed in phosphate buffer solution (PBS) and the ex vivo assays using the trade kiwifruit pollen artificially contaminated with a Psa Biovar 3, the CRA-FRU 8.43 strain. Biovar 3 is highly aggressive and was the phytopathogen responsible for the global pandemic of kiwifruit firstly reported in Italy in 2008.

## 2. Materials and Methods 

To evaluate the potential of aPDT with NMB and MB in the photo-inactivation of Psa and to select the best photo-inactivation conditions to be used in the pollen disinfection, in vitro assays were performed. The first experiments in vitro were performed in phosphate-buffered saline (PBS), using NMB at different concentrations (1.0, 2.5, and 5.0 μM), without and with KI addition (an aPDT potentiator), selected according to our previous studies [26,27] at a concentration of 100 mM. In a second step, the best in vitro aPDT conditions established for NMB were extended to MB (concentration 5.0 μM) and the assays were performed with and without the addition of 100 mM of KI. Then, the best tested PS, the MB, was used in the ex vivo assays, using trade kiwifruit pollen (containing its natural microorganisms) and trade kiwifruit pollen artificially contaminated with Psa (containing its natural microorganisms and the added Psa). In these ex vivo assays, the MB concentration was increased to 50 μM, according to the results obtained in our previous studies [9,23]. The natural microorganisms present on the kiwifruit pollen (total viable count, fungi, and bacterial endospores) were quantified in the trade pollen samples by viable plate count, and the Schaeffer–Fulton endospore stain was also performed. To evaluate the efficiency of pollen contamination with Psa, which was used in ex vivo assays, the Psa concentration was determined in the pellet (obtained by centrifugation from a Psa overnight culture) used to contaminate the pollen sample and in the pollen samples before the aPDT experiments. Lastly, the pollen germination ability was evaluated following aPDT treatment.

### 2.1. Bacterial Strain and Growth Conditions

The *Pseudomonas syringae* pv. *actinidiae* strain CRA-FRU 8.43 (Psa 3, also referred to as Psa V or Biovar 3) isolated in Lazio, Italy, in 2008, and obtained from the Culture Collection of the Centro di Ricerca per la Frutticoltura (Rome, Italy) [28,29] was used. The bacterial strain was grown in Luria–Bertani Agar (LA, Liofilchem, Roseto TE, Italy) at 25 °C for 48 h and then kept at 4 °C. Before each assay, a colony of the bacteria was aseptically inoculated in 30 mL of Tryptic Soy Broth (TSB, Liofilchem, Roseto TE, Italy) and grown aerobically for 24 h at 25 °C under stirring (120 rpm). The viable cell density was approximately 10^8^ colony-forming units per mL (CFU mL^−1^).

### 2.2. Kiwifruit Pollen 

The kiwifruit trade pollen was kindly supplied by the Portuguese Association of Kiwifruit growers (APK, Associação Portuguesa de Kiwicultores, Santa Maria da Feira, Portugal). The pollen obtained in dried form was stored at −20 °C in Falcon tubes duly sealed with Parafilm™.

#### Quantification of Pollen Natural Microorganisms and Endospore Stain

The natural microorganisms of pollen—total viable count, fungi cells, and bacterial endospores—were quantified by plating the pollen suspensions (prepared in PBS) in LA and Rose–Bengal Chloramphenicol Agar (RBCA) (Merck KGaA, Darmstadt, Germany). For that, two aqueous suspensions of 10 mg of pollen in 500 μL PBS were prepared. One of these suspensions was ten-folded diluted (10^0^ to 10^−3^) in PBS and 100 μL of each dilution were spread-plated in LA and RCBA media. The LA plates were incubated at 25 °C for 42 h and the RBCA plates at 25 °C for 5 days. The other suspension was heated at 80 °C for 10 min before plating in LA to determine the number of bacteria forming endospores. After the incubation periods, the number of CFU was counted on the most appropriate dilution. Three independent assays were performed for each different condition.

The presence of bacteria forming endospores was also evaluated by observing pollen samples under the optical microscope. One hundred microliters of the non-heated pollen suspension (non-diluted-10^0^) was spread-plated in Nutrient Agar (NA) (Merck KGaA, Darmstadt, Germany) enriched with 10 mg/L of manganese sulfate (MnSO_4_) and the plate was incubated for 3 days at 25 °C. The Schaeffer–Fulton endospore stain method [30] was used to distinguish between the vegetative cells and the endospores present on the pollen. The malachite green was used to stain the endospores and the safranin was used to stain the vegetative cells. The prepared slides were then observed under the optical microscope at 1000× total magnification (Upright Microscope-ECLIPSE 80i equipped with Digital Sight DS-U3 for data acquisition and Nikon Digital Sight DS-Ri1 camera (Tokyo, Japan)).

### 2.3. aPDT Assays

#### 2.3.1. Photosensitizers 

**Figure 1:**
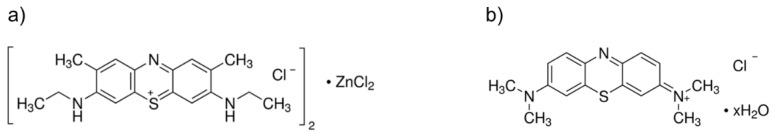
Chemical structures of the photosensitizers used in the study: (**a**) New Methylene Blue; and (**b**) Methylene Blue.

A stock solution (500 μM) of New Methylene Blue (New Methylene Blue N zinc chloride double salt; C_18_H_22_ClN_3_S 0.5 ZnCl_2_; IUPAC name: dichlorozinc ethyl-[7-(ethylamino)-2,8-dimethylphenothiazin-3-ylidene]azanium chloride; CAS 6586-05-6; dye content 90%; Sigma-Aldrich^®^/Merck KGaA, Darmstadt, Germany) (Figure 1a) was prepared in dimethyl sulfoxide (DMSO). A stock solution (500 μM) of Methylene Blue (C_16_H_18_ClN_3_S xH_2_O; IUPAC name: 3,7-bis(dimethylamino)phenazathionium chloride; CAS 122965-43-9; dye content ≥ 82%; Sigma-Aldrich^®^/Merck KGaA, Darmstadt, Germany) (Figure 1b) was prepared in PBS. Both stock solutions were wrapped in aluminum foil and stored in glass flasks in the dark, being sonicated in an ultrasonic bath for 15 min before each assay.

#### 2.3.2. Light Sources

**Figure 2:**
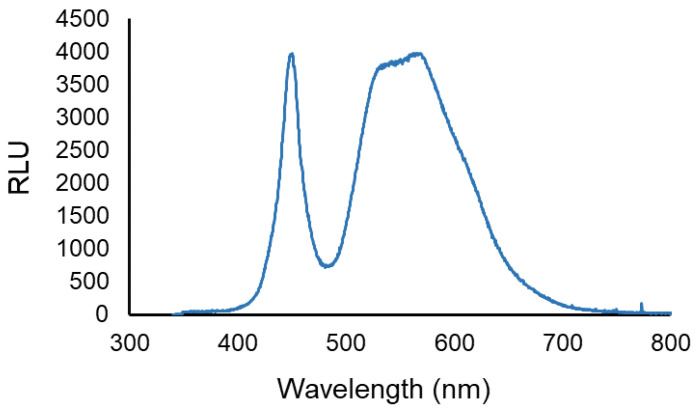
LED output. The spectral range of the white light emitted by the LED projector used. Data are shown as relative light units vs. wavelength.

The artificial white light was provided by a LED projector, (EL^®^MARK, power, voltage, and frequency of 20 W, ~230 V and ~50 Hz, respectively). The spectral range of the white light emitted by the LED projector is shown in Figure 2. The light irradiance was measured and adjusted to 50 mW cm^−2^ with the aid of a power and energy meter (model FieldMaxII-Top from Coherent, Santa Clara CA, USA) connected to a high-sensitivity sensor (model PS19Q, Coherent).

#### 2.3.3. In Vitro aPDT Assays with NMB and MB

A bacterial suspension (overnight inoculum with 10^9^ CFU mL^−1^ diluted tenfold in PBS to a final concentration of ≈10^8^ CFU mL^−1^) was prepared and 5.0 mL were distributed in Petri plates (φ = 40 mm) with the NMB added to a final concentration of 1.0, 2.5, or 5.0 μM. The Petri plates were incubated in the dark for 10 min at room temperature under magnetic stirring (100 rpm), to promote the PS binding to the cells. Light and dark controls were also prepared: the light control (LC) contained only the bacterial suspension; the dark control (DC) contained the bacterial suspension incubated with the PS at the studied concentration but was protected from light with aluminum foil. Sample and LC were irradiated under white light at an irradiance of 50 mW cm^−2^ for 90 min; the DC was kept in the dark for the same period of aPDT treatment. Aliquots of the sample, LC, and DC were collected at time 0 (after the pre-incubation period and immediately before irradiation) and after 5, 15, 30, 45, 60, and 90 min of irradiation, serially diluted in sterile PBS and 3 droplets of 5.0 μL of each dilution were plated in LA. The plates were then incubated at 25 °C for 48 h and the number of CFU was counted on the most appropriate dilution on the agar plates. 

Similar assays were performed using the same NMB concentrations (1.0, 2.5, and 5.0 μM) with the addition of KI (at a concentration of 100 mM). In these experiments, besides the LC, two other controls were included: a LC + KI (with KI at 100 mM) and a DC (NMB + KI) (with the NMB at the studied concentration and KI at 100 mM maintained in the dark).

The assays with MB were done only at the concentration of 5.0 μM with and without the addition of KI at a final concentration of 100 mM.

Three independent assays in triplicate were performed for each different condition. 

#### 2.3.4. Ex Vivo aPDT Assays with MB in Kiwifruit Pollen 

aPDT assays were performed with kiwifruit pollen containing its natural microorganisms (not contaminated artificially with Psa, hereafter designated as non-contaminated pollen) and in kiwifruit pollen containing its natural microorganisms and artificially contaminated with Psa. 

For the assays with non-contaminated pollen, 10 mg of pollen were weighed into an Eppendorf, added with 500 μL of sterile PBS, and serially diluted (10^0^ to 10^−5^) in PBS. 

Petri plates (φ = 40 mm) containing 40 μL of MB at 50 μM, spread on top of LA, were prepared in duplicate, one to be used at the beginning of the assay (*T*_0_, not irradiated), and the other to be irradiated (*T*_F_). Light and dark controls were prepared simultaneously: to the light control (LC), 40 μL of sterile PBS were spread on the surface of LA (without addition of MB); the dark control (DC) was prepared in the same way as the sample (with 40 μL of MB at 50 μM spread on top of LA plate) but was wrapped with aluminum foil to avoid the light incidence. These plates were divided into hexants (labeled 10^0^ to 10^−5^) and two 5.0-μL droplets of each dilution of pollen suspension were plated into the corresponding hexant. A pre-incubation of 1 h in the dark was made, to allow the PS binding to the bacterial cells. The sample and LC plates were irradiated with white light at an irradiance of 50 mW cm^−2^ for 180 min, whereas DC plate was kept protected from light for the same period. The plates were then incubated at 25 °C for 48 h and the number of CFU was counted on the most appropriate dilution on the agar plates. Three independent assays in duplicate were performed.

Similar assays were performed using KI as a potentiator agent. A solution containing MB at the concentration of 50 μM and KI at 100 mM was prepared and 40 μL of this solution were spread on the top of two LA plates-one to be irradiated (MB + KI) and the second one to be the dark control (DC (MB + KI)), which was protected from light during the irradiation period. Three independent assays with two technical replicates per assay were performed.

The same procedure was used for the aPDT assays performed with the kiwifruit pollen artificially contaminated with Psa. The assays were also performed with MB in the absence and the presence of KI.

To contaminate the pollen with Psa, 5.0 mL of an overnight Psa culture were centrifuged (5 min at 5000 rpm), the supernatant discarded, and the pellet resuspended in 1.0 mL of PBS. This bacterial suspension was added to 50 mg of pollen, gently homogenized via inverting and swirling, and incubated for 30 min at room temperature. The resulting mixture was then poured into sterilized standard filter paper and left to dry out in a laminar flow chamber for ca. 15 min. Subsequently, the pollen was scrapped off the filter paper, weighed again, and 10 mg were suspended in 500 μL of PBS, serial diluted (10^0^–10^−6^) and then plated using the procedure used in the aPDT experiments with the non-contaminated pollen.

The success of the artificial contamination of pollen with Psa was also evaluated before the aPDT experiments. A suspension of 10 mg of non-contaminated pollen and a suspension of 10 mg of artificially contaminated pollen were both prepared in 500 μL of PBS, serially diluted (10^0^–10^−8^), and three 5.0-μL droplets of each dilution were plated in LA. The resuspended pellet resulting from the centrifugation of the overnight Psa inoculum that was used to artificially contaminate the pollen was similarly diluted and plated. All the LA plates were incubated at 25 °C for 48 h and the number of CFU was counted on the most appropriate dilution on the agar plates. 

**Figure 3:**
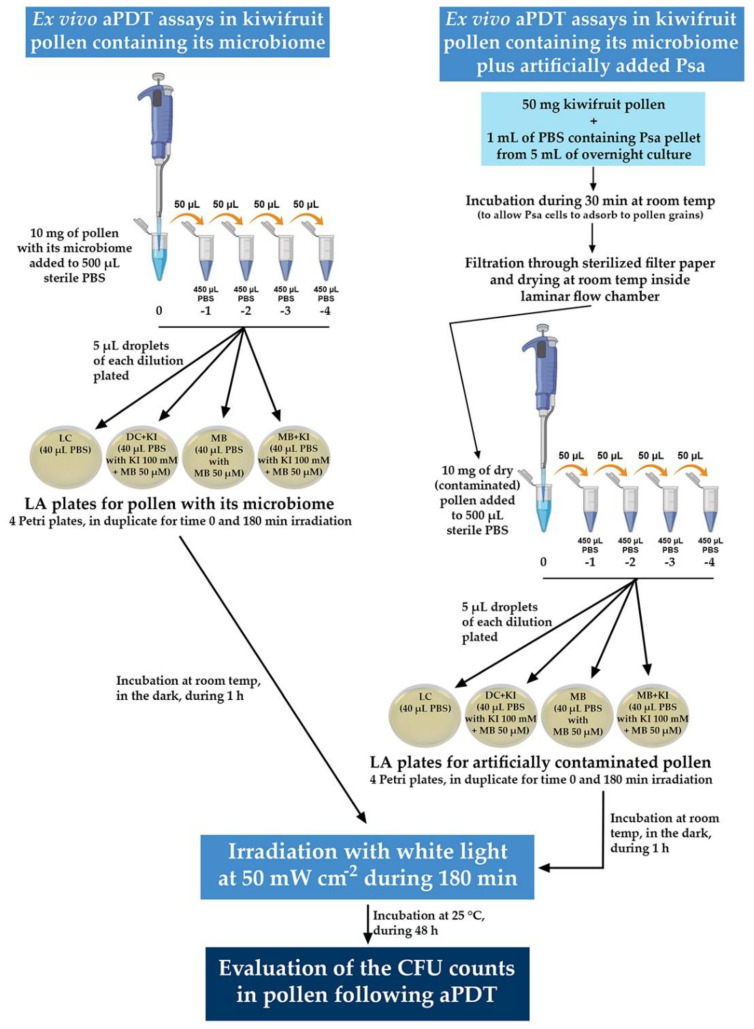
Schematic representation of the experimental procedure followed for the ex vivo aPDT assays. All the condition plates (LC, DC + KI, MB, and MB + KI) were made in duplicate to use one set for time 0 and the other set for time 180 min.

The schematic representation of the experimental procedure followed for the ex vivo aPDT assays is depicted in detail in Figure 3. 

### 2.4. Evaluation of Pollen Germination after aPDT

To assess if the pollen viability was affected after aPDT, the pollen germination ability was evaluated. For this, 5.0 mg pollen were weighed into Eppendorf tubes and added with 200 μL PBS. To the pollen sample (MB), 40 μL of MB at 50 μM were added; to the sample added with KI (MB + KI), 40 μL of a solution containing MB at 50 μM and KI at 100 mM were added. Light and dark controls were also prepared: to the light control (LC), 40 μL of PBS were added; to the dark control (DC (MB + KI)), 40 μL of the same solution prepared with MB at 50 μM and KI at 100 μM were added. The resulting suspension mixtures were homogenized by gently inverting and swirling, after which they were transferred into the corresponding well on 12-well cell culture plates. All the samples were prepared in duplicate: one for *T*_0_ (without irradiation) and the other to be irradiated (*T*_F_). LC, MB, and MB + KI were then irradiated with artificial white light at an irradiance of 50 mW cm^−2^ for 180 min. DC (MB + KI) was protected from light via wrapping with aluminum foil.

After aPDT treatment, to each well in the plates from the previous step were added 1.20 mL of pollen germination medium (KCl 100 mg/L, H_3_BO_3_ 100 mg/L, CaCl_2_ 300 mg/L, MgSO_4_ 200 mg/L, sucrose 2.5%, PEG 10%, pH 7.5) and the plates were incubated at 25 °C for 24 h under gentle orbital shaking. After the incubation period, three microscope slides were prepared for each suspension using 100 μL aliquots of the suspension and observed under 100× total magnification (Upright Microscope-ECLIPSE Ni-U equipped with CoolLED pE-300-W, Digital Sight DS-U3 for data acquisition and Nikon Digital Sight DS-Qi1Mc camera (Tokyo, Japan)). For each slide, at least 25 photomicrographs were gathered, amounting to a total of 75 photomicrographs for each suspension/well. Pollen germination was evaluated by calculating the percentage of germinated pollen grains out of a total number of pollen grains in the 75 photomicrographs.

**Figure 4:**
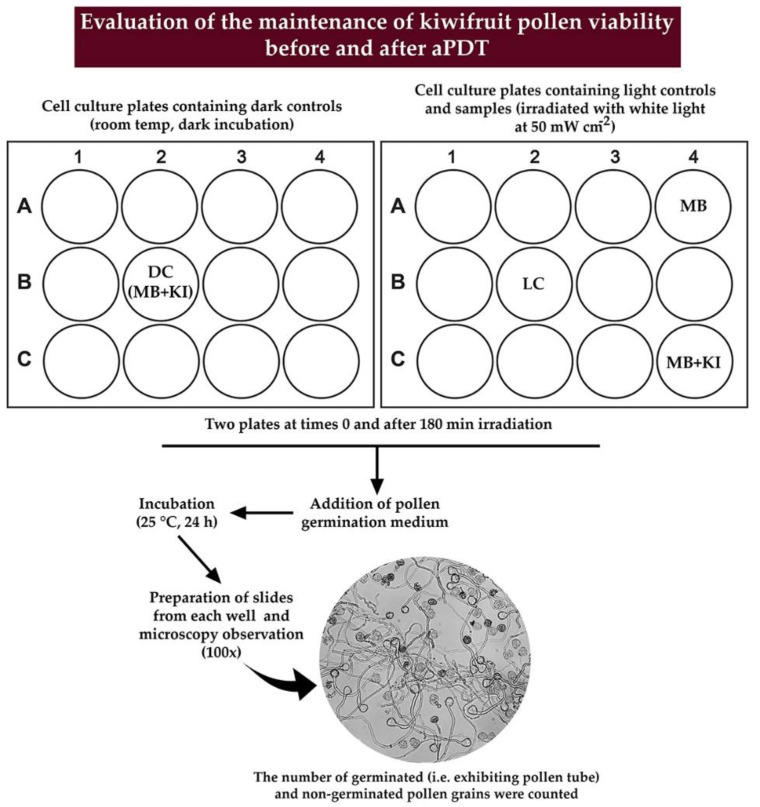
Schematic representation of the experimental procedure used to evaluate the pollen germination after aPDT.

The schematic representation of the experimental procedure followed for evaluating the pollen germination is depicted in detail in Figure 4.

### 2.5. Statistical Analyses

Statistical analysis of the data was carried out using the GraphPad Prism 7.04 (GraphPad Software, San Diego, CA, USA). The normal distribution of the data was checked by a Kolmogorov–Smirnov test and the homogeneity of variance was assessed by the Brown–Forsythe test. For a pairwise comparison of the means, Tukey’s multiple comparison test was used. The significance of bacterial concentrations between treatments, and along the experiments, was tested using two-way ANOVA and Dunnet’s multiple comparison tests were applied to assess the significance of the differences between the tested conditions. For different treatments, the significance of differences was evaluated by comparing the results obtained in the test samples after treatment with the results obtained for the correspondent test samples before treatment. For all cases, at least three independent assays in duplicate were performed. In the case of ex vivo aPDT assays with MB in kiwifruit pollen, six independent assays were performed using MB alone. A *p* < 0.05 value was considered to be statistically significant.

## 3. Results

### 3.1. Quantification of Pollen Natural Microorganisms and Endospore Stain

**Figure 5:**
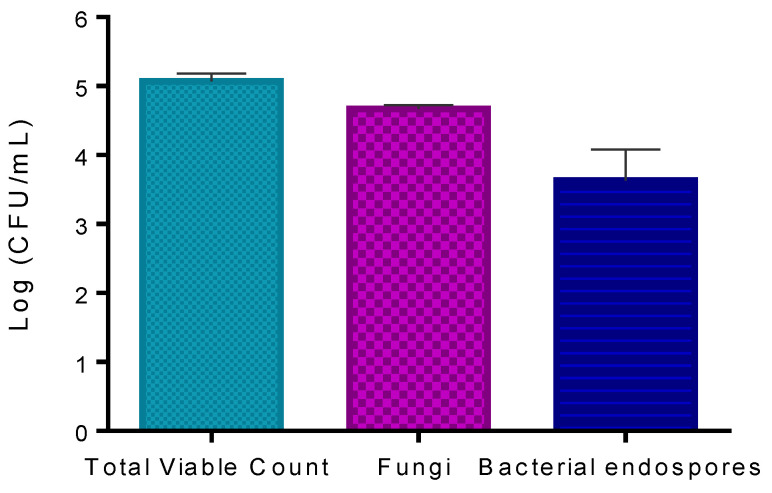
Quantification of natural microorganisms on trade pollen: total viable count, fungi, and bacterial endospores. Values represent the mean of three experiments; error bars represent the standard deviation.

The results obtained show that the concentration of viable microorganisms present on kiwifruit pollen counted in LA was 5.1 log CFU mL^−1^ (Figure 5). The number of fungi counted in RBCA was 4.7 log CFU mL^−1^. The number of bacterial endospores counted in LA after heating the sample to 80 °C for 10 min was 3.6 log CFU mL^−1^.

**Figure 6:**
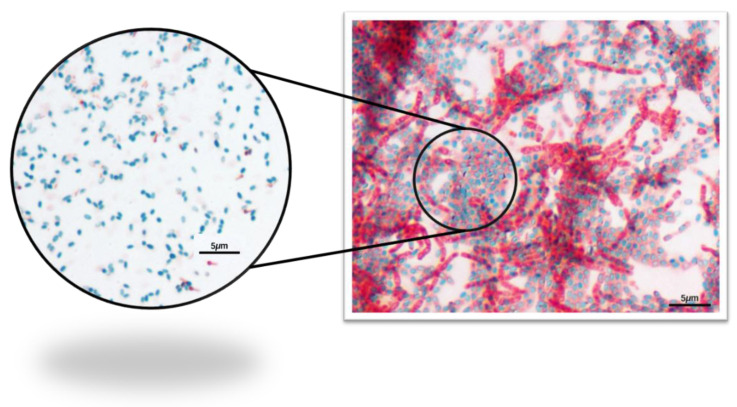
Photomicrographs of the pollen Schaeffer–Fulton endospore stain—1000× total magnification.

After the Schaeffer–Fulton endospore stain, the presence of endospores (as green ellipses) was visualized under microscopy (Figure 6).

### 3.2. In Vitro aPDT Assays with NMB and MB

**Figure 7:**
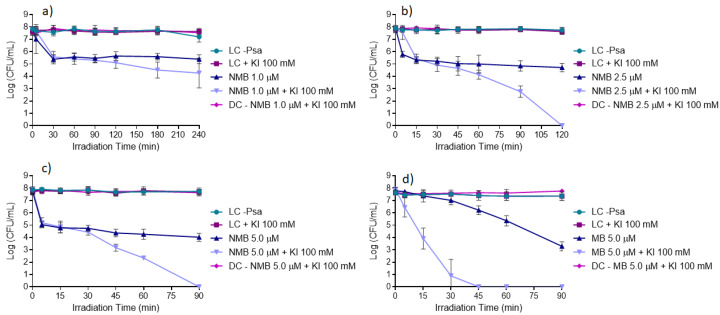
In vitro inactivation of Psa CRA-FRU 8.43 via aPDT, using NMB at: (**a**) 1.0 μM; (**b**) 2.5 μM; and (**c**) 5.0 μM; and (**d**) MB at 5.0 μM as photosensitizers, in the absence and presence of KI (at 100 mM). Values represent the mean of three independent assays in triplicate; error bars represent the standard deviation. LC, light control; DC, dark control. Lines just combine the experimental points.

The results obtained from the in vitro inactivation assays using NMB at 1.0, 2.5, and 5.0 μM with and without KI (Figure 7a–c) show that the highest reductions of Psa were achieved with NMB in the presence of KI compared to the ones achieved with the NMB alone. The results also show that NMB at 5.0 μM with the presence of KI at 100 mM (Figure 7c) was the best tested condition to inactivate Psa, allowing the total inactivation of the bacteria (ca. 8 log CFU mL^−1^ (ANOVA, *p* < 0.05)) after 90 min of white light irradiation at an irradiance of 50 mW cm^−2^. This PS at 5.0 μM in the absence of KI promoted a significantly lower reduction of bacterial concentration, with a maximum of ca. 3.7 log CFU mL^−1^ (ANOVA, *p* < 0.05) reduction. When NMB was used at the concentration of 2.5 μM and combined with KI, the total photo-inactivation of Psa was achieved after 120 min of irradiation (Figure 7b). With NMB at the lowest tested concentration (1.0 μM), a reduction of only ca. 3.6 log CFU mL^−1^ (ANOVA, *p* < 0.05) in the bacterial concentration was achieved even in the presence of KI and after 240 min of light irradiation (Figure 7a).

The photodynamic efficiency of MB was also studied using the most efficient NMB conditions (5.0 μM with the addition of KI at 100 mM) and the results represented in Figure 7d show that the combination of MB with KI was more effective than NMB with KI, attaining the same reduction of ca. 8 log CFU mL^−1^ (ANOVA, *p* < 0.05) in bacterial concentration (total inactivation) in just half of the irradiation time (45 min). Therefore, MB was selected for the subsequent ex vivo aPDT assays in kiwifruit trade pollen.

It is also important to refer that all the controls performed, i.e., LC, light control; LC + KI, light control with KI; and DC (PS + KI), dark control with PS + KI, remained stable during the whole experiment, indicating that Psa viability was not affected by light (LC) itself, as well as neither by the PS alone nor PS plus KI in the absence of light (DC (PS + KI)).

### 3.3. Ex Vivo aPDT Assays on Kiwifruit Pollen with MB

#### 3.3.1. Photo-Inactivation of Pollen Natural Microorganisms

**Figure 8:**
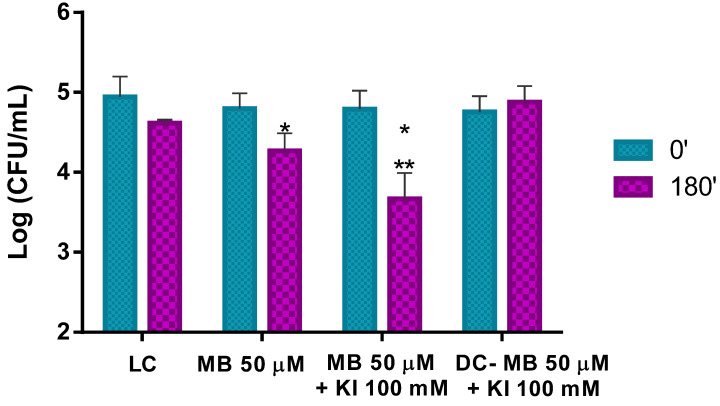
Ex vivo aPDT assay of pollen natural microorganisms, irradiated with 50 mW cm^−2^ for 180 min, using MB at 50 μM and MB at 50 μM with the addition of KI (at 100 mM). Values represent the mean of three independent assays; error bars represent the standard deviation. * *p* < 0.05 (relative to each condition at time 0′); ** *p* < 0.05 (relative to MB). LC, light control; DC, dark control.

The results obtained in the ex vivo aPDT assays performed with MB at 50 μM with non-artificially contaminated pollen (containing only its natural microorganisms) and irradiated for 180 min with white light at an irradiance of 50 mW cm^−2^ show that aPDT treatment induces a reduction of only 0.5 log CFU mL^−1^ (ANOVA, *p* < 0.05) in the viability of the natural microorganisms after the irradiation protocol (Figure 8). When the MB combined with KI was used in the aPDT treatment of non-artificially contaminated pollen, a significant decrease of ca. 1.1 log CFU mL^−1^ (ANOVA, *p* < 0.05) in the natural microorganisms’ survival (Figure 8) was obtained. Light (LC) and dark (DC (MB + KI)) controls did not show statistically significant reductions. Comparing the effect of MB with and without KI in the photo-inactivation of natural microorganisms of trade pollen, it was possible to see that MB + KI generated a decrease of more 0.6 log CFU mL^−1^ in the viability of the natural microorganisms compared to MB alone, showing the potentiator effect of KI.

#### 3.3.2. Photo-Inactivation of Artificial Psa Contaminated Pollen

**Figure 9:**
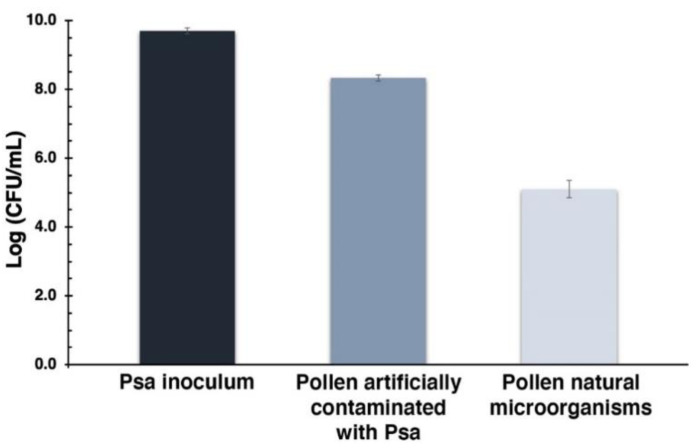
Efficiency of artificial pollen contamination with Psa. The first bar represents the Psa inoculum used to contaminate the pollen; the bar in the middle represents the total contamination achieved; the last bar corresponds to the pollen natural microorganisms. Values represent the mean of three independent assays in triplicate; error bars represent the standard deviation.

To evaluate the aPDT action in Psa present on the kiwifruit pollen, the trade pollen was previously contaminated with Psa to be assured that this bacterium was also present on pollen besides its own natural microorganisms (Figure 9).

The Psa inoculum used to contaminate the pollen had a concentration of 9.7 log CFU mL^−1^, leading to a total of 8.3 log CFU mL^−1^ on pollen (which corresponds to pollen natural microorganisms plus added Psa). This value is clearly above pollen natural microorganisms’ concentration, which is 5.1 log CFU mL^−1^. These results indicate that ca. 3.2 log CFU mL^−1^ of Psa was added to pollen.

**Figure 10:**
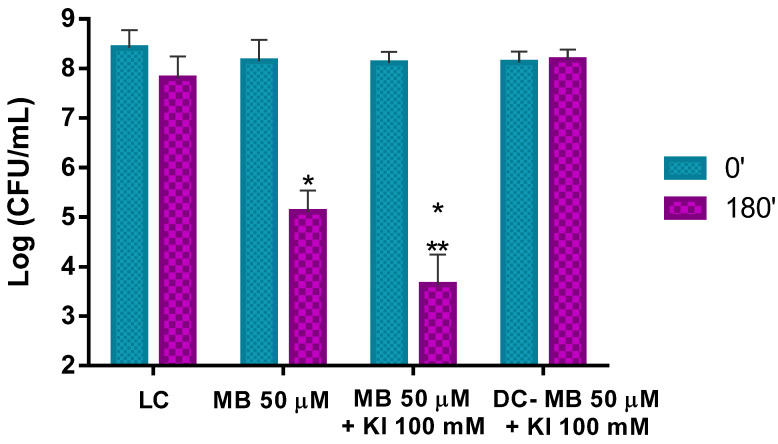
Ex vivo aPDT assay on pollen containing its natural microorganisms plus artificial Psa contamination, irradiated with 50 mW cm^−2^ for 180 min, using MB at 50 μM and MB at 50 μM with the addition of KI at 100 mM. Values represent the mean of three independent assays; error bars represent the standard deviation. * *p* < 0.05 (relative to each condition at time 0′); ** *p* < 0.05 (relative to MB). LC, light control; DC, dark control.

This pollen containing its natural microorganisms and artificially contaminated with Psa was then subjected to aPDT using MB at 50 μM alone and MB at the same concentration combined with KI at 100 mM and the results are presented in Figure 10. The results show a remarkable aPDT effect performed by MB and MB + KI in the photo-inactivation of Psa on kiwifruit pollen. When MB was used alone, a reduction of ca. 3.1 log CFU mL^−1^ (ANOVA, *p* < 0.05) of bacterial survival was achieved after the white light irradiation period of 180 min at an irradiance of 50 mW cm^−2^.

When aPDT treatment was carried out with the combination of MB at 50 μM and KI at 100 mM, a decrease of ca. 4.5 log CFU mL^−1^ (ANOVA, *p* < 0.05) was observed under the same irradiation conditions. This corresponds to an additional significant decrease of ca. 1.4 log CFU mL^−1^ (ANOVA, *p* < 0.05) when comparing the results with the PS alone, showing, again, the potentiator effect of KI (Figure 10).

In addition, in this case, light and dark controls did not show statistically significant reductions.

### 3.4. Evaluation of Pollen Germination after aPDT

**Figure 11:**
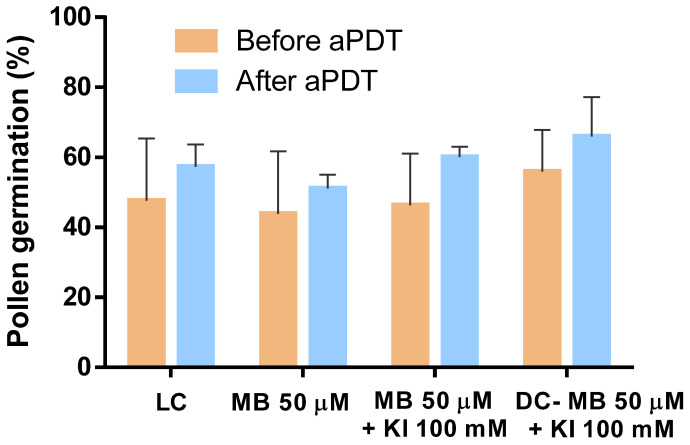
Evaluation of pollen germination before and after aPDT. Values represent the mean of pollen germination percentage resulting from three independent assays in triplicate; error bars represent the standard deviation.

The evaluation of pollen ability to germinate following aPDT assays was performed by assessing the capability of pollen grains to germinate before and after being subjected to aPDT. The results obtained and statistically analyzed clearly indicate that aPDT has no significant detrimental effect on pollen germination, with none of the samples reducing its germination percentage (Figure 11). The sample treated with MB exhibited a pollen germination of 44% and 51% before and after aPDT, respectively. The sample treated with MB combined with KI exhibited pollen germination of 46% and 60% before and after aPDT, respectively.

Light and dark controls also showed that pollen percentages of germinations did not suffer reduction when submitted to aPDT.

## 4. Discussion

Kiwifruit pollen has been recognized as an important source and vector of Psa dissemination, hence elimination of Psa contamination on kiwifruit pollen is of utmost importance for the kiwifruit production sector. While there are no antimicrobial treatments currently available for use in pollen, research is slowly progressing towards the development of potentially effective, while environmentally safe, antimicrobial approaches for this high-added value commodity. In the literature, only one study specifically related to the treatment of pollen using heat [25] was found. Two other recent studies have demonstrated that aPDT can be used to successfully control Psa on kiwifruit leaves [9,23], which prompted to think that aPDT could be an efficient alternative to inactivate Psa from kiwifruit pollen.

Buffered solutions, such as PBS, are useful to evaluate the action and efficacy of the PSs in a medium without organic matter and cell interference to select the best PS and the more appropriate aPDT conditions. However, as the composition of the test matrix is important in aPDT, to pave the way for real application, it is required to test the PSs in relevant settings, such as the case of this study in pollen.

Firstly, in vitro assays were performed with NMB at three different concentrations (1.0, 2.5, and 5.0 μM) with and without KI (at 100 mM). These assays showed that, when using KI as a potentiator agent, significantly higher reductions of the bacteria survival were achieved when comparing with the ones obtained with the PS alone. It was also observed that the PS at 5.0 and 2.5 μM in the presence of KI allowed total inactivation (ca. 8 log CFU mL^−1^ reduction) of the bacteria, but the highest concentration led to a significantly higher reduction of the time needed to inactivate the Psa (90 min vs. 120 min of irradiation, respectively) (Figure 7b,c).

As MB is a widely known PS due to its antimicrobial effects [31], the best conditions of photo-inactivation with NMB (PS at 5.0 μM and with the addition of KI at 100 mM) were used for the PS MB (at the same concentration). A Psa total inactivation was also obtained, but, when KI was added, only half of the time was needed to inactivate the Psa (45 min) (Figure 7d).

Previous aPDT studies already showed that MB was an efficient PS in the inactivation of various microorganisms, namely other Gram-positive bacteria such as *Staphylococcus aureus*, Gram-negative bacteria such as *Pseudomonas aeruginosa* [32] and *Vibrio parahaemolyticus* [33], and fungi such as *Colletotricum abscissum* [34] and *Candida albicans* [35].

Moreover, aPDT with other PS, i.e., Tetra-Py(+)-Me porphyrin lead to a Psa decrease of 6 log in the in vitro assays after 60 min under an irradiance of 4.0 mW cm^−2^ and a decrease of 1.8 log CFU mL^−1^ with an irradiance of 150 mW cm^−2^ in the ex vivo assays using kiwi leaves [9]. Other studies have also shown a 7.4 log CFU mL^−1^ Psa decrease in vitro after 60 min of irradiation (4.0 mW cm^−2^) on the presence of a formulation constituted by five cationic porphyrin derivatives and a 2.8 log CFU mL^−1^ decrease on artificially contaminated leaves with the same formulation and light irradiance [23].

The potentiator effect of KI was extensively studied by several groups, and it was demonstrated that KI reacts with ^1^O_2_, affording free iodine (I_2_/I_3_^−^), hydrogen peroxide (H_2_O_2_), and iodine radicals (I_2_^•−^), which are extremely bactericidal [26,36,37,38,39,40,41,42,43]. In fact, several in vitro and in vivo studies have shown the potentiation of fullerenes, Rose Bengal, and MB by KI towards several microorganisms such as *Acinetobacter baumannii*, *P. aeruginosa*, *C. albicans,* and methicillin-resistant *S. aureus* [36,37,40,42,43]. The results obtained in vitro in the present study and comparing with previous ones prompted us to evaluate if MB was efficient for photo-inactivation assays ex vivo, i.e., in kiwifruit pollen.

Since the ex vivo aPDT assays were performed in trade pollen that already contained its natural microorganisms, the pollen natural microorganisms were first quantified (Figure 5). As pollen is not a nutrient-rich matrix and water availability is low, suggesting the growth of some bacteria but also of some fungi and some sporulating bacteria, the bacterial, fungal, and bacterial endospore concentrations on the pollen grains were determined. A microbial concentration of around 5 log CFU mL^−1^ was determined in LA (a non-selective medium), which allows mainly the growth of bacteria, but also of some fungi after 48 h of incubation [44]. The fungal concentration was also determined in RBCA (a fungi selective medium) after five days of incubation. A fungal concentration of 4.7 log CFU mL^−1^ was determined. In addition, 3.6 log CFU mL^−1^ of bacterial endospores was observed in the heated pollen suspension incubated in the LA. The Schaeffer–Fulton stain allowed visualizing the high number of endospores (Figure 6). The addition of the manganese sulfate to the culture medium promoted the bacterial sporulation, confirming the existence of endospore-forming bacteria on the pollen. These results (Figure 5 and Figure 6) show that the microorganisms naturally present on pollen are abundant and that some less aPDT susceptible microorganisms (bacterial endospores and fungi) relatively to Psa (a non-sporulating Gram-negative bacterium) are present in higher concentrations [45,46]. In fact, the aPDT treatments performed with MB at 50 μM with non-contaminated pollen were almost ineffective against the natural microorganisms of pollen (Figure 8). Even with the addition of KI (at 100 mM), a decrease of only 1.1 log CFU mL^−1^ (ANOVA, *p* < 0.05) was observed (Figure 8), which can be justified by the high concentration of fungi and endospores present on the pollen.

Since the main goal of this work was to evaluate the efficiency of aPDT to inactivate Psa on kiwifruit pollen, the non-Psa contaminated pollen was artificially contaminated with Psa. A protocol to artificially contaminate the provided pollen was efficiently developed, allowing to contaminate the pollen with around 3.2 log CFU mL^−1^ of Psa. As the natural microorganisms’ content was about 5.1 log CFU mL^−1^, a total of 8.3 log CFU mL^−1^ of microorganisms was present on the artificially contaminated pollen (Figure 9). When the artificially contaminated pollen was submitted to aPDT in the presence of MB alone, a reduction of 3.1 log CFU mL^−1^ (ANOVA, *p* < 0.05) was achieved after 180 min of white light irradiation (Figure 10). An additional decrease of 1.4 log CFU mL^−1^ (ANOVA, *p* < 0.05) was observed when KI was used as a potentiator agent, with a total reduction of 4.5 log CFU mL^−1^ (ANOVA, *p* < 0.05), which corresponds to an effective Psa inactivation. These results also demonstrate the potentiator effect of KI.

As the Psa concentration of the artificially contaminated pollen was ca. 3.1 log CFU mL^−1^, it is possible to conclude that almost all the Psa cells added artificially (ca. 3.2 log CFU mL^−1^) to the pollen were inactivated by aPDT, whereas aPDT had a minimal effect upon natural microorganisms of pollen.

According to the American Society of Microbiology, the minimum reduction required for a new approach to be termed as an antimicrobial is at least 3 log CFU mL^−1^, which corresponds to a reduction of 99.9% of bacterial concentration (ASM, 2015), thus the aPDT protocol used has proved to be an efficient approach, even without KI, to inactivate Psa on kiwifruit pollen.

Knowing that aPDT efficiency could not be dissociated from the maintenance of pollen ability to germinate, its ability to germinate was also evaluated under controlled conditions before and after the aPDT treatment. The results show that the aPDT treatment did not negatively affect the germination (Figure 11).

The results of this study show that the easily accessible photosensitizer used in this study (MB) is an excellent approach to photo-inactivate Psa on pollen and the protocol designed and used may be feasible to be used in the treatment of kiwifruit pollen naturally contaminated with Psa, which has been proven to be responsible for the dissemination of Psa among orchards [24]. An additional positive outcome of this antimicrobial technology is the fact that a minimal effect on the natural microorganisms of pollen was observed, which may be a positive aspect for aPDT application in the environment, since non-pathogenic microorganisms present on pollen may not be too affected. Actually, a recent study showed that the kiwifruit microbiota may contain biocontrol strains against Psa. Some *Streptomyces* strains showed not only the ability to colonize both kiwifruit rhizosphere and phyllosphere, but also an effective antibacterial activity. The antibacterial activity was associated with the production of anti-Psa secondary metabolites, through enzymatic ability, and with the presence of antibiotic gene clusters in their genome [47].

The field application of aPDT can be similar to that already tested ex vivo for the kiwifruit leaves [9,23], that is, by spraying the kiwifruit pollen with the PS and using white light LEDs as light source. However, to translate the application of this strategy into practice, more studies are needed, namely using sunlight irradiation after pollen application in orchards.

Overall, the extensively studied PS, MB, allows an effective photo-inactivation of *Pseudomonas syringae* pv. *actinidiae* on kiwifruit pollen under white light irradiation without imparting any significant damage to the trade pollen features.

## 5. Patents

A patent has resulted from the work reported in this article. Adelaide Almeida, Margarida M. Lopes, Maria Bartolomeu, M. Amparo F. Faustino, M. Graça P. M. S. Neves, Ana T. P. C. Gomes, Bruno Pinto, Luís Reis. *Use of photodynamic therapy to control the phytopathogenic Pseudomonas syringae* pv. *actinidiae agent in kiwi pollen*. PN patent application number 116297, filled 25 April 2020.
